# A Novel Tb@Sr-MOF as Self-Calibrating Luminescent Sensor for Nutritional Antioxidant

**DOI:** 10.3390/nano8100796

**Published:** 2018-10-07

**Authors:** Yi Wang, Shaomin Lin, Jun Luo, Rui Huang, Hong Cai, Wei Yan, Huan Yang

**Affiliations:** 1 College of chemistry and Material Engineering, Gui Yang University, Guiyang 550005, China; wangy49@mail2.sysu.edu.cn (Y.W.); 2243@hstc.edu.cn (J.L.); caolm3@mail2.sysu.edu.cn (W.Y.); 2 School of Material science and Engineering Han Shan Normal University, Chaozhou 521041, China; lsm678@hstc.edu.cn (S.L.); rhuang@hstc.edu.cn (R.H.); glcai@hstc.edu.cn (H.C.)

**Keywords:** Tb(III) functionalized, metal organic framework, self-calibrating and reusable luminescent sensor, sesamol

## Abstract

Sesamol, is well-known antioxidant and can reduce the rate of oxidation and prolong expiration date. It is also potentially antimutagenic and antihepatotoxic, the detection of sesamol is important and remains a huge challenge. Herein, a new 3D alkaline earth Sr metal organic framework [Sr(BDC)·DMAC·H_2_O]n (BDC = benzene-1,4-dicarboxylate; DMAC = *N*,*N*-dimethylacetamide) is synthesized and a probe based on Tb^3+^ functionalized Sr-MOF. The Tb(3+)@Sr-MOF showed good luminescence and thermal property. Due to the energy competition between sesamol and ligand, the luminescence intensity of sesamol increases meantime luminescence intensity of Tb^3+^ decreases, the ratio of the emission intensities (I_344_/I_545_) linearly increases with sesamol in concentrations ranging from 1 × 10^−7^ to 8 × 10^−4^ M. Furthermore, the fluorescence-detected circular test shows that the composite Tb(3+)@Sr-MOF can serve as ratiometric sensor for sensing of sesamol. This is the first example for self-calibrated detecting sesamol based on metal-organic framework (MOF).

## 1. Introduction

Sesame oil is a high-priced, high-quality health food that is popular in China and India because it contains a number of bioactive phytochemical; it is very high in natural antioxidants in the form of lignans. Antioxidant compounds in sesame seed oil that are beneficial impacts on health have attracted increasing attention. Sesamol (3,4-methylenedioxyphenol) is a natural phenolic lignan found in sesame seed or sesame oil as well as has shown promising antioxidant and neuroprotective effects [1,2]. Recently, tremendous research has shown that sesamol can weaken injury in endotoxemic rats, lower serum lipids and blood pressure; it also has potentially anti-hypertensive and anti-inflammatory activities in humans. Sesamol content plays an important role human health and the flavor of sesame oil, therefore there is a need for sesamol determination for evaluate lignan content in sesame oil or other food, meanwhile, determination of sesamol in different environment with high selectivity and sensitivity has also become a major research topic. Some detection methods for sesamol have been developed [3,4] such as high-performance liquid chromatography (HPLC) or ultraviolet (UV) detection ultraviolet (UV) detection. However, many disadvantages have various limitations such as time-consuming, cost, complicated preparation process, and the need for professionals. Therefore, it is exigent to explore a kind of simple, rapid, highly selective, and sensitive access for detecting sesamol [5].

Metal-organic frameworks (MOFs), are crystalline porous architectures that are composed of metal ions or clusters and organic ligand, have been emerging as very promising materials which can be applied in various fields due to their unique properties, such as high surface areas, controllable pore size. MOFs have been proposed to be promising in many applications, such as gas storage and separation [6,7,8,9,10,11], catalysis [12,13,14,15], energy storage [16,17], and biomedical applications [18,19,20,21,22,23]. Among luminescent MOFs, Ln-MOFs have huge advantages in sensing due to their fascinating luminescence characteristics, such as high luminescence quantum yield, long luminescent life time, high color purity, and big stoke shifts. The prominent luminescent properties and various structures of Ln-MOFs provide a facile and easy method for detection [24,25,26] such as metal ions [27,28], anions [29,30,31,32], small molecule [33,34,35,36], and temperature [37,38]. Due to the high coordination number and multiple coordination modes of lanthanide ions, the design and preparation of the desired lanthanide MOF is also a huge challenge. Poststsynthetic modification method of MOFs provide an effective tool to perform the fluorescence sensing properties of lanthanide MOFs by introduction of Ln^3+^ into MOFs, which can exploit and expand their application [39,40,41]. In comparison to the reports on other metal organic framework, alkaline earth metals have been rarely reported, but alkaline earth metals have applied in many field of materials science because of some advantages (low-cost, avirulent). Based on aforementioned reasons, alkaline earth salts are usually used as commercial materials, which are extensively used in daily life and industry production, such as common medicament, dyes, and pigments. Some studies about alkaline earth metal complexes should be performed. Mg and Al based MOFs exhibit excellent hydrogen storage ability because of low density, high surface areas, and controlled structures [42,43].

In addition, MOF sensors depends on the fluorescent intensity of single emission which usually influenced by many uncontrollable factors such as drift of light source and sensor concentration. Therefore, dual-emissive luminescent probe should be one breakthrough because this type of signal by making a comparison of the emission intensities of two different luminescent center to form self-calibrating mechanism that can avoid external factor such as fluctuations of light source, voltage, show improved sensitivity, and to the intended analyte [44,45,46,47]. However, there is no report for self-calibrating MOF-based luminescent probe that can selectively detect antioxidant sesamol molecule.

Herein, according to the above considerations and our previous work on Ln-metal organic frameworks [48,49], a new 3D Sr metal-organic framework is designed and synthesized. The Tb(3+)@Sr-MOF were successfully synthesized via encapsulating Tb^3+^ in Sr-MOF. When energy competition exists between sesamol and ligand, which blocks the energy transfer from ligand to Tb^3+^, hence the luminescent intensity of sesamol at 330 nm increases and Tb^3+^ ion at 545 nm decreases. A linear relationship between the ratio of luminescent intensities (I_343_/I_545_) and the concentration of sesamol. Thus, this composite Tb(3+)@Sr-MOF can be served as a promising self-calibrating sensor for sesamol sensing.

## 2. Experimental Section

### 2.1. Synthesis of [Sr(BDC)·DMAC·H_2_O]n (Sr-MOF)

A mixture of SrCl_2_ (0.1 mmol, 15.7 mg) and H_2_BDC (0.1 mmol, 16.6 mg) were stirred in 3 mL DMAC and added in a 15 mL Teflon cup and heated to 80 °C for 72 h then cooled to room temperature. The colorless block single crystals were obtained, rinsed with DMAC and H_2_O, and dried in air (56% yield based on BDC).

### 2.2. Synthesis of Tb(3+)@Sr-MOF

Powder of Sr-MOF (100 mg) is soaked in Tb (NO_3_)_3_·6H_2_O ethanol solution (10 mL, 2 mmol) for 1 day. The powder is isolated by centrifugation and washed with ethanol three times, the obtained product dried in air for 24 h. The metal amount of Tb:Sr is 1:22.

### 2.3. Detection of Sesamol

The obtained Tb(3+)@Sr-MOF (2.00 mg) dispersed in 4 mL ethanol and ultrasonicated for 5 min. Different concentrations of sesamol ethanol solutions were prepared and mixed with suspension of Tb(3+)@Sr-MOF for the detection of sesamol. For the selectivity of sesamol detection, 1 × 10^−3^ M for sesamol, 4-Methylcatechol, catechol, guaiacol, carvacrol, paeonol, thymol, vanillin, resorcinol, and 1,3-dichlorophenol were also prepared and added to each of the suspension of Tb(3+)@Sr-MOF, respectively. In order to examine the cycle performance of Tb(3+)@Sr-MOF, the suspension is formed by dispersing the sample (1 mg/mL) into ethanol. After detection of sesamol, the suspensions of Tb(3+)@Sr-MOF/sesamol are obtained by filtration and rinsed several times with ethanol, then the Tb(3+)@Sr-MOF was dried naturally and ready for the next cyclic test.

### 2.4. Materials and Characterization

Sesamol, 4-Methylcatechol, catechol, guaiacol, carvacrol, paeonol, thymol, vanillin, resorcinol, 1,3-dichlorophenol, terbium nitrate and Strontium chloride were purchased from Mackin (Macklin. Shanghai. China). The metal ion content in Tb(3+)@Sr-MOF(Tb:Sr) were examined using inductively coupled plasma mass spectrometry ICP-MS, Icap Qc (Thermo-Fisher, Massachusetts, MA, USA). X-ray diffraction (XRD) patterns were characterized using a Rigaku Miniflex 600 X-ray diffractometer (Rigadu, Tokyo, Japan) from 5° to 50°. All the emission spectra for the Tb(3+)@Sr-MOF were recorded by Horiba ihr320 fluorescence spectrophotometer(Horiba, Kyoto, Japan). An infrared spectrum was recorded and taken on a IR Affinity-1 FT-IR spectrometer (Shimadzu, Kyoto, Japan) in the range of 400–4000 cm^−1^. Thermogravimetric analysis (TGA) results were measured from 50 °C to 700 °C under nitrogen atmosphere on a Netzsch sta 449f3 (Netzsch, Bararia, Germany).

### 2.5. X-ray Crystallography

Crystal of Sr-MOF was collected from the mother liquor. Single-crystal data of Sr-MOF were collected on a Rigaku Oxford CCD diffractometer equipped with graphite-monochromatic Mo-K α radiation (λ = 0.71073 Å) at 293 K. The structure was solved by direct methods, and refined by full-matrix least-square method with the SHELX-2016 program package. The crystallographic data and refinements and the selected bond lengths and angles for Sr-MOF are listed in Table 1 and Table 2.

## 3. Results and Discussion

**Figure 1:**
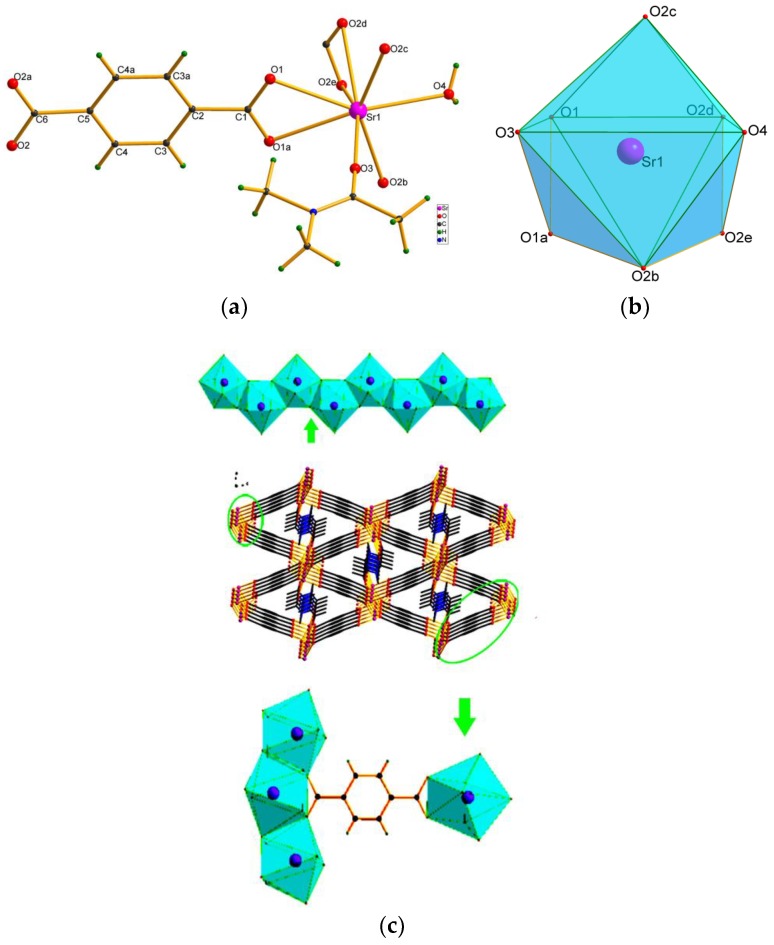
(**a**) Asymmetric unit of Sr-MOF. (**b**) Coordination mode of the Sr^2+^ ion. Atoms marked with are at the following symmetry positions: a = x, 1.5 − y, z; b = 0.5 − x, 1 − y, −0.5 + z; c = 0.5 − x, 0.5 + y, −0.5 + z; d = −0.5 + x, 1.5 − y, 0.5 − z; e = −0.5 + x, y, 0.5 − z. (**c**) Packing diagram of 1 projected along the c axis, showing the chains of bicapped octahedra and the connectivity between them.

The crystals of Sr-BDC belong to the orthorhombic space group Pnma, the asymmetric unit is made up of half a Sr^2+^ ion, half a BDC^2−^ ligand, half a DMA, and half a water molecules (Figure 1a). The Sr^2+^ ion is bound to eight O atoms from one H_2_O, one DMAC and four BDC^2−^ ligands, which form an octahedron which adopted distorted bicapped coordination. The DMAC and H_2_O are monodentate, and the COO^−^ of a BDC^2−^ adopted two coordination modes with Sr^2+^ ions: η1:η1 and η2:η2—bridging mode, which link one and three Sr^2+^ ion. In Figure 1b, in the bicapped octahedron, the Sr-O bond distance vary from 2.490(6) to 2.687(5) Å. A zigzag chain is formed by adjacent octahedra along the b axis, and the chains are connected by the BDC^2−^ (μ4,η1:η1:η2:η2-bridging mode) forming a three-dimensional framework, (Figure 1), which form quadrangular channel (two kinds of triangular channels), DMAC molecules are filled and connected directly to the Sr^2+^ ions in the channels.

**Figure 2:**
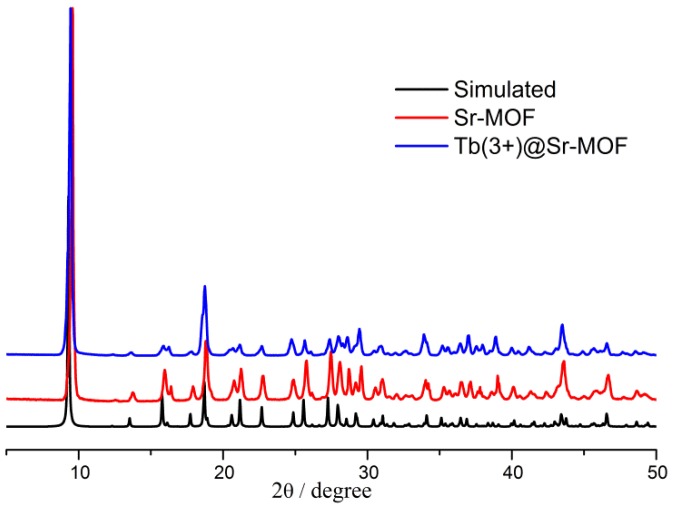
PXRD patterns of the simulated Sr-MOF, Tb(3+)@Sr-MOF.

The XRD patterns of simulated and as-synthesized Sr-MOF, Tb(3+)@Sr-MOF are shown in Figure 2. All the diffraction peaks (the Sr-MOF and Tb(3+)@Sr-MOF) were well corresponded to those in the simulated PXRD pattern of Sr-MOF(CCDC:1551141). The introduction of Tb^3+^ will not influence the crystal form of Sr-MOF.

**Figure 3:**
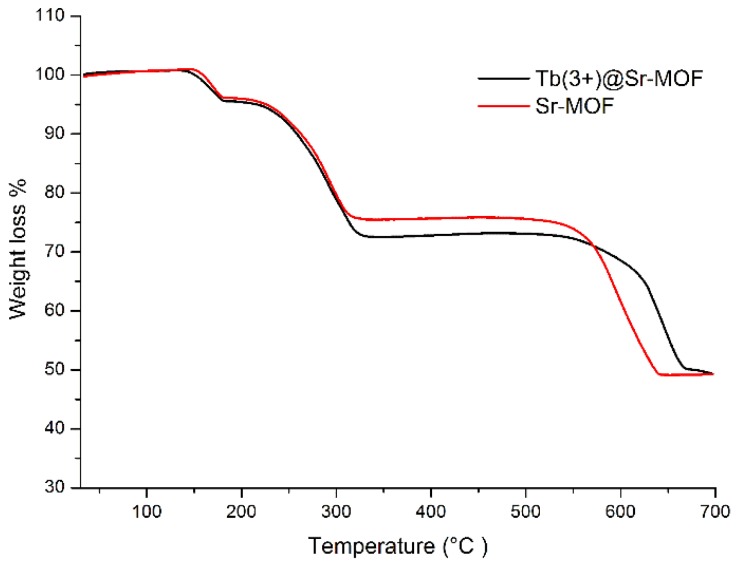
TGA of Sr-MOF and Tb(3+)@Sr-MOF.

As shown in Figure 3, the TG measurement show that Tb@Sr-MOF and Sr-MOF similar thermal stability and exhibit three events of mass (Tb@Sr-MOF and Sr-MOF) reduction. The TG curve shows that Tb@Sr-MOF and Sr-MOF starts to reduce mass at ~130 °C due to the removal of water molecules and complete dehydration is at about 200 °C. The second plateau of reducing mass start from 200 °C to 310 °C corresponds to the loss of DMAC. The decomposition of the organic ligand begins at 580 °C and ends at 630 °C. The final stage of reducing mass start from 630 °C corresponds to oxide.

**Figure 4:**
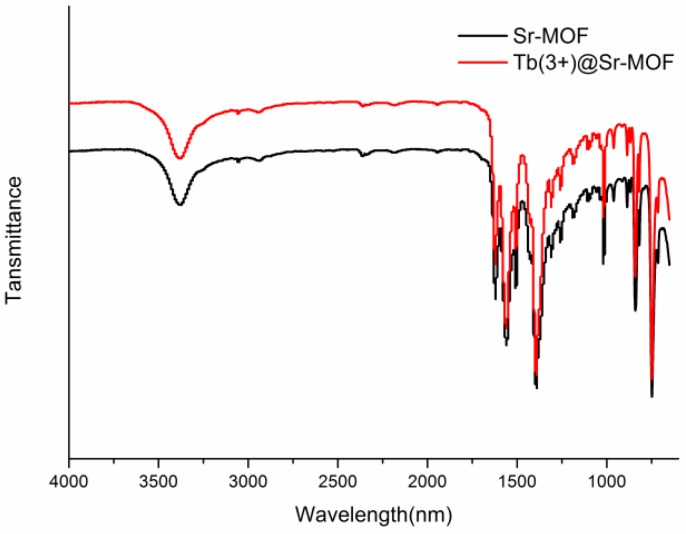
IR spectra of Sr-MOF and Tb(3+)@Sr-MOF.

As shown in Figure 4, the broad band at 3300−3500 cm^−1^ is assigned to the characteristic stretching vibrations of O−H in H_2_O. The peak at 1560 cm^−1^ belongs to υ_C-O_. After incorporating Tb^3+^ into the Sr-MOF, the absorption band of Tb(3+)@Sr-MOF agrees with those of Sr-MOF. The result shows that the introduction of Tb^3+^ does not affect the crystalline integrity, as shown in Figure 2.

**Figure 5:**
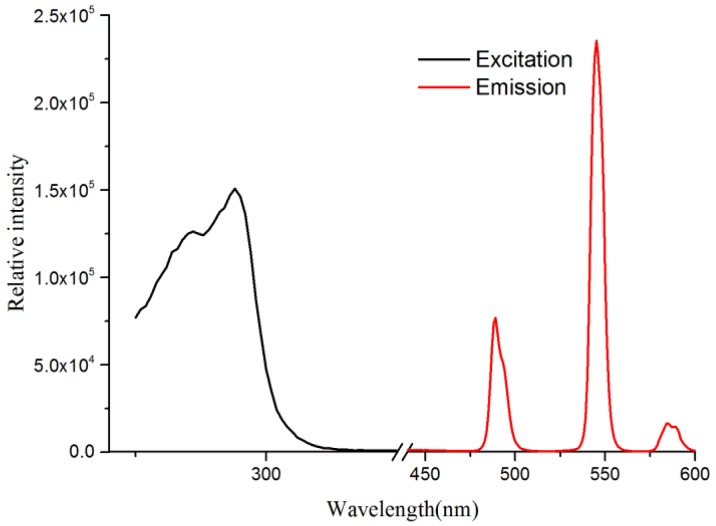
The excitation (dashed) and emission (solid) spectra of the Tb(3+)@Sr-MOF.

As seen in Figure 5, Tb(3+)@Sr-MOF exhibits characteristic emission of the Tb^3+^ ion when excited 294 nm. Tb(3+)@Sr-MOF exhibits three peaks at 489, 545, and 592 nm originated from ^5^D_4_→^7^F_J_ (J = 6, 5, 4) transitions, respectively. The emission bands of the Tb(3+)@Sr-MOF at 545 nm show a bright green light. The results suggested Tb(3+)@Sr-MOF can act as a luminescence sensor.

**Figure 6:**
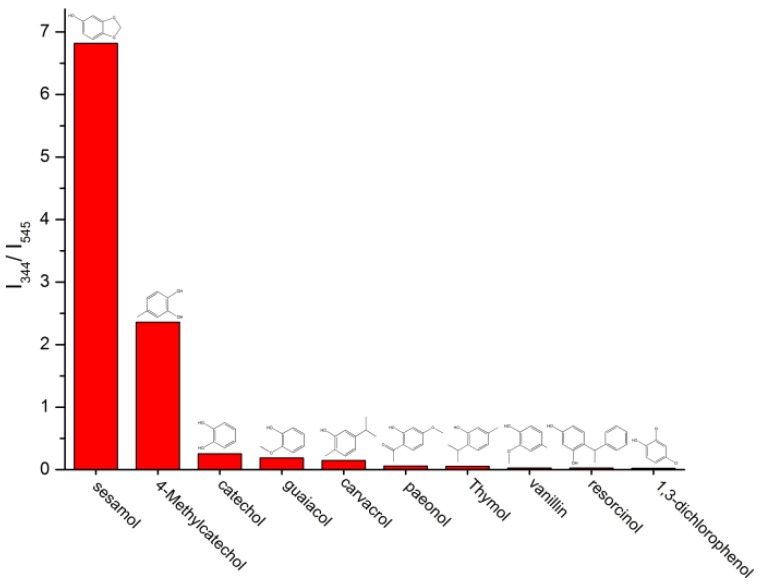
Diagrams of the transition intensities (I_344_/I_545_) of the Tb@Sr-MOF in various small biomolecules (λex = 294 nm).

The sensing ability of the Tb(3+)@Sr-MOF was investigated in the presence of different molecules. As shown in Figure 6, on the addition of 1 × 10^−3^ M of biomolecules (sesamol, 4-Methylcatechol, catechol, guaiacol, carvacrol, paeonol, thymol, vanillin, resorcinol, and 1,3-dichlorophenol), however, the luminescence intensity of Tb(3+)@Sr-MOF at 545 nm exhibit the strongest luminescence quenching in the presence of sesamol, meantime, and luminescent intensity (the emission at 330 nm) increases significantly with the increasing the concentration of sesamol, We speculate that the emission spectrum at 330 nm is ascribed to sesamol.

**Figure 7:**
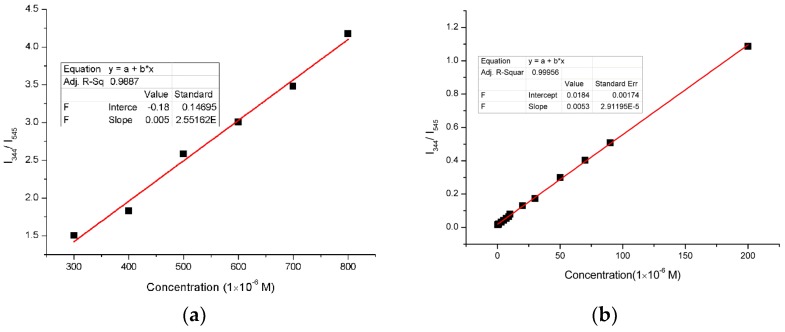

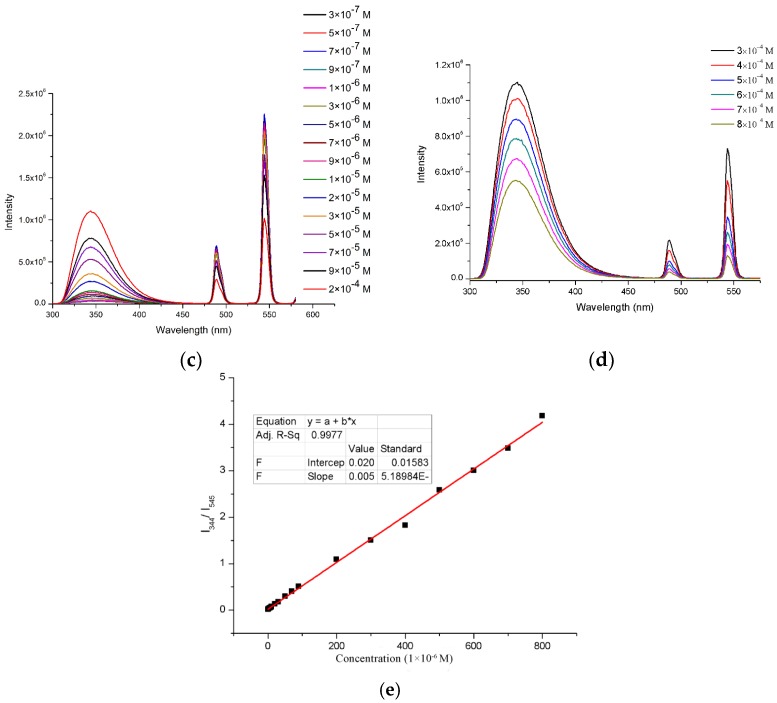
(**a**) Plot of I_344_/I_545_ versus the sesamol content (1 × 10^−7^ to 2 × 10^−4^ M) in ethanol solution (λ_ex_ = 294 nm); (**b**) Plot of I_344_/I_545_ versus the sesamol content (3 × 10^−4^ to 8 × 10^−4^ M) in ethanol solution; (**c**) Emission spectra of Tb(3+)@Sr-MOF as a function of the sesamol concentration (from top: 1 × 10^−7^ to 2 × 10^−4^ M) in ethanol solution; (**d**) Emission spectra of Tb(3+)@Sr-MOF as a function of the sesamol concentration (3 × 10^−4^ to 8 × 10^−4^ M) in ethanol solution; (**e**) Plot of I_344_/I_545_ versus the sesamol content (1 × 10^−7^ to 8 × 10^−4^ M) in ethanol solution (λ_ex_ = 294 nm).

In order to overcome such disadvantages of the traditional single emission sensing, Tb(3+)@Sr-MOF were synthesized and could be served as ratiometric luminescent sensor for sesamol. In Figure 7, the change of luminescent intensity (Tb(3+)@Sr-MOF) displayed with a concentration of sesamol increases. The luminescent intensity at 330 nm increased meantime the fluorescence intensity of Tb^3+^ at 545 nm decreased. The plot of the luminescent intensity ratio I_330_/I_545_ against the concentration of added sesamol was shown in Figure 7a,b, the luminescent intensity ratio I_330_/I_545_ has a good linear relationship to the concentration of sesamol varying from 1 × 10^−7^ to 2 × 10^−4^ M and 3 × 10^−4^ to 8 × 10^−4^ M, which was described by calibrating function of I_330_/I_545_ = 0.00538 + 0.0184 × C and I_330_/I_545_ = 0.005 × Csesamol-0.18 with a correlation coefficient of 0.99966 and 0.9887. Interestingly, when the concentration of sesamol reaches 3 × 10^−4^ M, luminescent intensities of I_330_ and I_545_ decreases, respectively. The luminescent intensity ratio I_330_/I_545_ also has a good linear correlation to the concentration of sesamol in the range from 1 × 10^−7^ to 8 × 10^−4^ M calibrating function of I_330_/I_545_ = 0.02 + 0.005 × C sesamol with a correlation coefficient of 0.9977. The limit of detection (LOD = 3δ/S, δ represents the blank solution was measured ten times, and S stands for the slope of the calibration curve was about 4.2 μM [50]. The above results illustrated that Tb(3+)@Sr-MOF is an excellent candidate for self-calibrating luminescent sensor (sesamol) and is not influenced by environmental factors.

**Figure 8:**
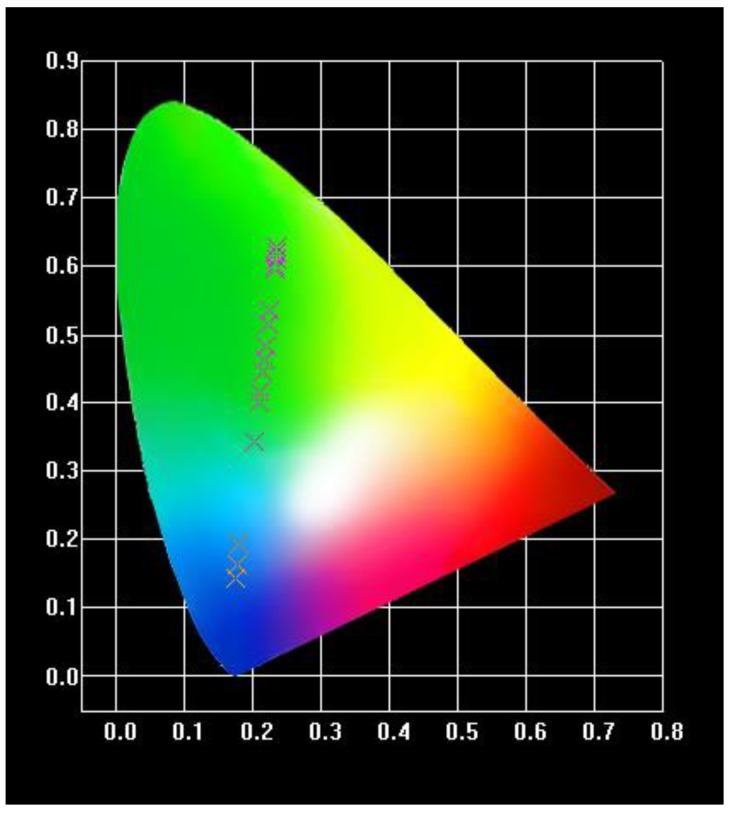
CIE chromaticity coordinates.

The CIE (Commission International deLEclairage) diagram of the Tb(3+)@Sr-MOF treated with different concentrations of sesamol was performed. As shown in Figure 8, luminescent color of Tb(3+)@Sr-MOF tuned from blue to green when excited at 294 nm. The results show that the luminescent ratio (I_344_/I_545_) is highly sensitive to the concentration of sesamol. The feature could be used served for sensing of different concentrations of sesamol with high selectivity and sensitivity and without any addition.

**Figure 9:**
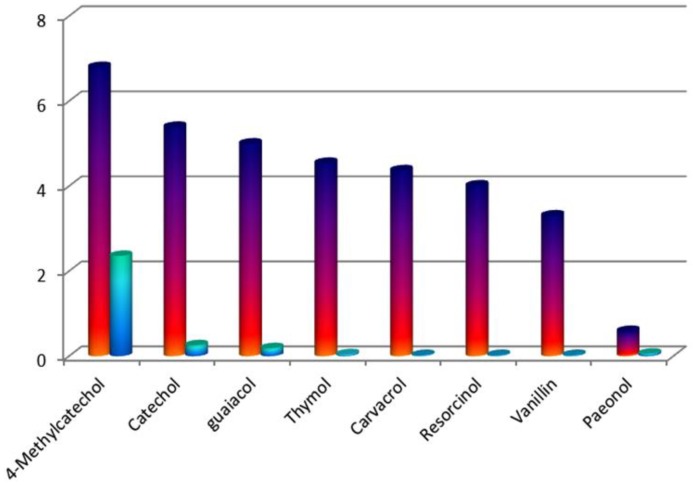
Luminescence responses of Tb(3+)@Sr-MOF to various biomolecules. The color bars suggest the relative ratio of luminescent intensities (I_344_/I_545_) treated with biomolecules. The blue bars suggest the relative ratio of I_344_/I_545_ treated with other biomolecules and sesamol) (λ_ex_ = 294 nm).

From a practical standpoint, the probe should have good response and high selectivity to the detecting. As seen in Figure 9, to access the selectivity of Tb(3+)@Sr-MOF, the competitive experiment was performed by adding 1 × 10^−3^ M sesamol to the Tb(3+)@Sr-MOF in the presence of 1 × 10^−3^ M other biomolecules (including 4-Methylcatechol, catechol, guaiacol, thymol, carvacrol, resorcinol, vanillin, and paeonol). The addition of biomolecules will not influence the changed trend of the ratio of I_330_/I_545_. (colorful columns in Figure 4), However, when added 1 × 10^−3^ M sesamol to the Tb(3+)@Sr-MOF containing other biomolecules, the luminescent intensity ratio I_330_/I_545_ increased remarkably (blue columns in Figure 4). Therefore, the results show that the Tb(3+)@Sr-MOF is a reliable and high-efficient self-calibrating sensor for sesamol.

**Figure 10:**
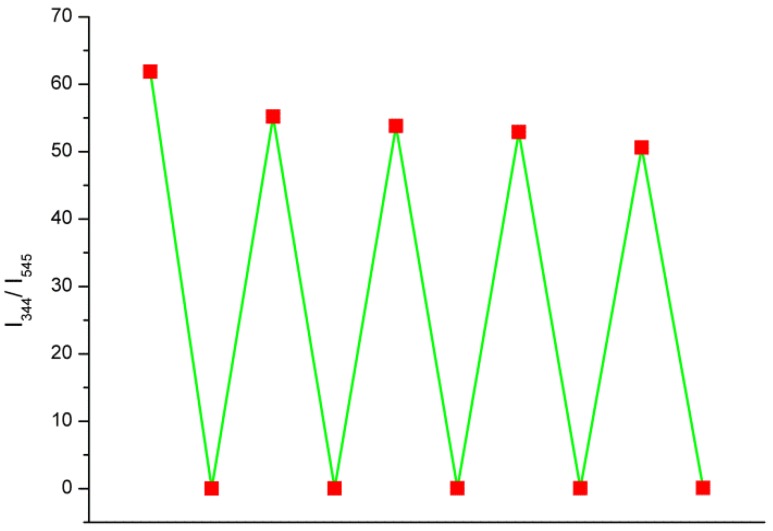
Cyclic response of luminescence intensities (I_344_/I_545_) of the Tb(3+)@Sr-MOF for detecting sesamol.

Furthermore, the cycling ability is an important indicator to access the sensor’s practicability. The Tb(3+)@Sr-MOF can be reused five times (Figure 10). After five cycles, the results show that the luminescence intensity of the recycled Tb(3+)@Sr-MOF almost agrees with those of the initial Tb(3+)@Sr-MOF, Meanwhile, These results reveal that Tb(3+)@Sr-MOF displays well reusability of sensing sesamol, suggesting its practical use in sesamol detection.

**Figure 11:**
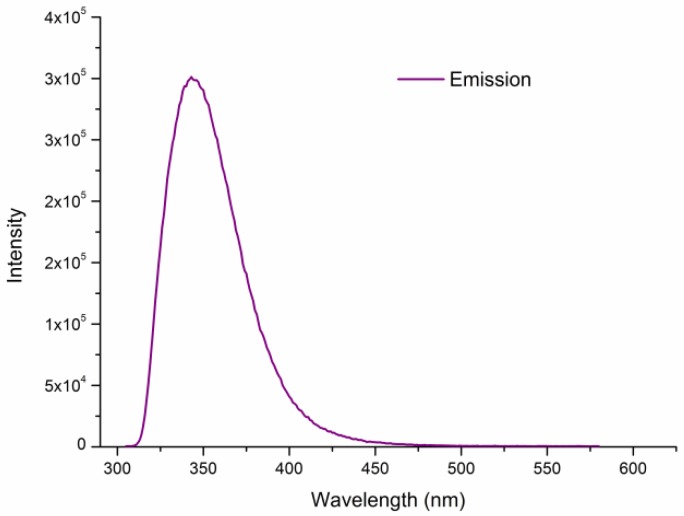
The emission (solid) spectra of the sesamol (λ_ex_ = 294 nm).

**Figure 12:**
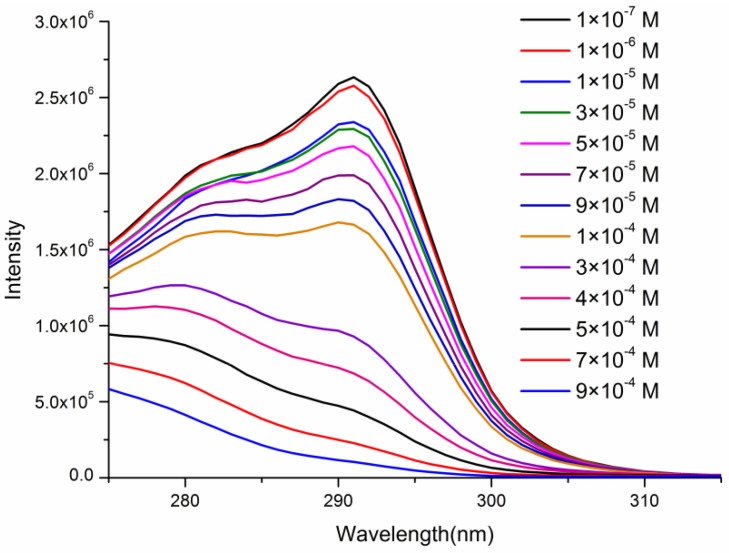
The excitation spectra of the Tb(3+)@Sr-MOF treated with sesamol.

While the quenching mechanism for biomolecules is still not very clear, it is necessary to study the possible quenching mechanism. (1) The emission spectra of sesamol was monitored when excited 294 nm, as shown in Figure 11, the I_344_ is consistent with I_344_ in Figure 7c,d. The result shows that luminescent signal(I_344_) in Figure 7c,d is assigned to sesamol. (2) As shown in Figure S1, the excitation spectra of the ligand within Tb(3+)@Sr-MOF is overlapped by the excitation spectra of sesamol, which suggests an excitation energy competition between the ligand and sesamol exists. Sesamol absorbs most of the energy and only a small fraction of energy will be transferred from the linker to the Tb^3+^ ions. The PXRD patterns of the Tb(3+)@Sr-MOF treated with sesamol reveal that its crystal structure is not changed and is consistent with the original Tb(3+)@Sr-MOF (as shown in Figure S2). (3) To better understand why luminescent intensities of I_344_ and I_545_ decreases when concentration of sesamol reached 3 × 10^−4^ M, we monitored the excitation spectra of Tb(3+)@Sr-MOF treated with various concentrations of sesamol under the monitoring wavelength(545 nm). As shown in Figure 12, with the increased concentration of sesamol, the intensities of excitation spectra of Tb(3+)@Sr-MOF decreases and a blue shift in the excitation maxima(294 to280 nm) could be observed for Tb(3+)@Sr-MOF treated with different concentrations of sesamol, leading to decline in luminescent intensity(I_344_ and I_545_), respectively, the fluorescence intensity ratio I_330_/I_545_ has also a good linear relationship to the concentration of sesamol vary from 1 × 10^−7^ to 8 × 10^−4^ M, The results suggested that Tb(3+)@Sr-MOF can serve as a self-calibrating luminescent sensor for sesamol and is not influenced by environmental factors.

## 4. Conclusions

In summary, a new 3D alkaline earth Sr metal organic framework is synthesized and chosen as a host to sensitize via encapsulating Tb^3+^ in Sr-MOF. Tb(3+)@Sr-MOF display excellent luminescent property and thermal stability. Due to energy competition between sesamol and ligand, the luminescent intensity of sesamol (I_344_) increases meantime luminescence intensity of Tb^3+^(I_545_) decreases. The Tb(3+)@Sr-MOF can be used as ratiometric sensor for sesamol. It is the first time reported that the rational design and preparation of luminescent MOFs for ratiometric sensing of sesamol relying on the ratio of emission-peak-height of analyte (sesamol) to lanthanide ions (Tb^3+^) as the detectable signals. In addition, this strategy may promote the development of lanthanide functionalized MOF for self-calibrating sensing and broaden the application of alkaline earth metal organic framework.

## Figures and Tables

**Table 1:** Crystal data and structure refinement for Sr-MOF.

Compound	Sr-MOF
Chemical Formula	C_12_H_15_NO_6_Sr
Formula weight	356.87
Crystal system	Orthorhombic
Space group	Pnma
*a* (Å)	10.9304 (3)
*b* (Å)	6.96110 (10)
*c* (Å)	19.0421 (4)
*α* (°)	90
*β* (°)	90
*γ* (°)	90
*V* (Å^3^)	1488.87
*Z*	4
*D_c_* (g/cm^3^)	1.636
*μ* (mm^−1^)	1.347
*T* (K)	293 (2)
Mo Kalpha	1.54178
*F* (000)	720
Crystal size (mm)	0.23 × 0.19 × 0.15
*θ* range (°)	4.64 to 67.08
Index ranges	−11 ≤ h ≤ 12
	−6 ≤ k ≤ 8
	−22 ≤ l ≤ 22
Reflections collected	6263
Independent reflections	1398[R_int_ = 0.0326]
Parameters	109
Goodness-of-fit on *F*^2^	1.112
*R*_1_ indices [*I* > 2*σ*(*I*)]	0.0340
*wR*_2_ indices [*I* > 2*σ*(*I*)]	0.0878
*R*_1_ indices [all data]	0.0369
*wR*_2_ indices [all data]	0.0902

**Table 2:** Bond lengths [A] and angles [deg] for Sr-MOF.

Main Coordination Modes Bond Length
Sr(1)-O(2)#1 2.490(2)
Sr(1)-O(2)#2 2.490(2)
Sr(1)-O(3) 2.496(4)
Sr(1)-O(4) 2.570(3)
Sr(1)-O(1) 2.642(2)
Sr(1)-O(1)#3 2.642(2)
Sr(1)-O(2)#4 2.689(2)
Sr(1)-O(2)#5 2.689(2)
Sr(1)-C(6)#4 2.971(5)
O(2)-Sr(1)#8 2.490(2)
O(2)-Sr(1)#9 2.689(2)
Symmetry transformations used to generate equivalent atoms:
#1 −x + 1/2, −y + 1, z − 1/2 #2 −x + 1/2, y + 1/2, z − 1/2
#3 x, −y + 3/2, z #4 x − 1/2, y, −z + 1/2 #5 x − 1/2, −y + 3/2, −z + 1/2
#6 −x, −y + 1, −z #7 −x, −y + 2, −z #8 −x + 1/2, −y + 1, z + 1/2
#9 x + 1/2, y, −z + 1/2

## Supplementary Materials

Materials and Methods are available online at http://www.mdpi.com/2079-4991/8/10/796/s1, Figure S1: The excitation spectra of sesamol(monitored wavelength 330nm), Figure S2: PXRD patterns of the Tb(3+)@Sr-MOF treated with sesamol (1 × 10^−2^ M).
