# Supplementary material for: A Novel Tb@Sr-MOF as Self-Calibrating Luminescent Sensor for Nutritional Antioxidant
Source: Nanomaterials (Basel). 2018 Oct 7;8(10):796. doi: 10.3390/nano8100796 (PMC6215301; doi:10.3390/nano8100796)
Supplement: Supplementary file 1 [file nanomaterials-08-00796-s001.pdf]

A Novel Tb@Sr-MOF as Self-Calibrating Luminescent Sensor for Nutritional Antioxidant

Yi Wang [2], Shaomin Lin [1], Jun Luo [2], Rui Huang [1], Hong Cai [1], Wei Yan [2] and Huan Yang [1,*]

[1] School of Material science and Engineering Han Shan Normal University, Chaozhou 521041, China; lsm678@hstc.edu.cn (S.L.); rhuang@hstc.edu.cn (R.H.); glcai@hstc.edu.cn (H.C.)

[2] College of chemistry and Material Engineering, Gui Yang University, Guiyang 550005, China; wangy49@mail2.sysu.edu.cn (Y.W.); 2243@hstc.edu.cn (J.L.); caolm3@mail2.sysu.edu.cn (W.Y.)

* Correspondence: yanghuan@hstc.edu.cn; Tel.: +86-076-8630-5234

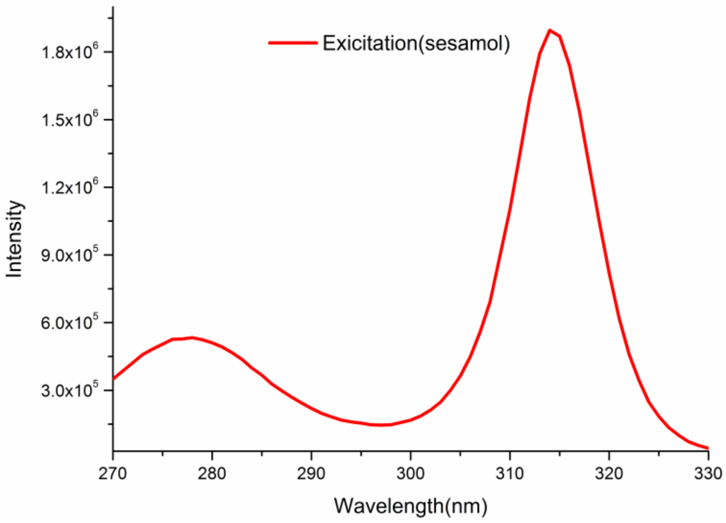

Figure. S1 The excitation spectra of sesamol(monitored wavelength 330nm).

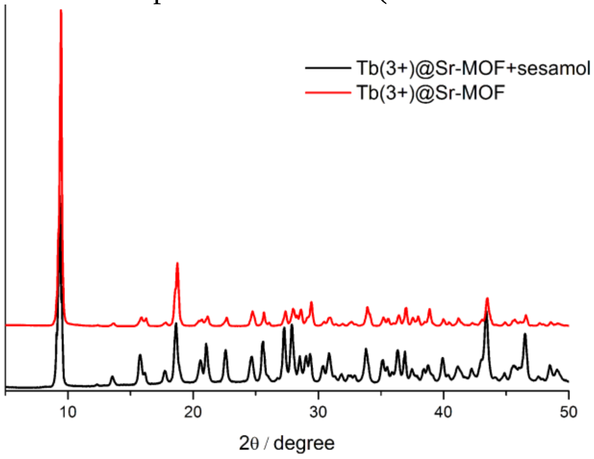

Figure. S2 PXRD patterns of the Tb(3+)@Sr-MOF treated with sesamol(1×10^{-2} M).
